# Rush Medical College Clinic

**Published:** 1877-05

**Authors:** Edmund M. Landis


### RUSH MEDICAL COLLEGE CLINIC.

### Service of PROF. MOSES GUNN.

(Reported by Edmund M. Landis. M. D.)

*Hare Lip and Cleft Palate, with Operation for the Hare Lip.*

Frederick W., German, aged 26, farmer, who is a robust young man, presents himself with a hare lip, and a completely fissured palate. The dental arches of the superior maxilla are even, and the teeth perfect and regular, up to the edges of the fissured bones, which approximate at the symphysis.

The hare lip has been operated upon, in Germany, unsuccessfully. Union after the operation, taking place only in the upper angle of the slit.

The ability of the patient to articulate, is illustrated by the following alphabetical score: a, b, —, —, e, f, g, —, i, j, —, l, m, n, o, — , (says u for g), r, —, —, u, v, w, x, y, —. The dashes represent the sounds which he is unable to master. The German h–hah, can be made, but the h–haitch, as in English, cannot be accomplished.

This case presents a complete cleft, but is a favorable one for an operation. It is believed that it could be operated upon successfully, though it is not proposed to recommend it, as we have seen but in a single instance, any improvement in articulation as a result of the operation. The habits of speech, or the mode of pronunciation, which a patient who has attained the age of this young man has acquired, are difficult to overcome. The pliability of the velum as seen in health, which cannot be obtained by an operation, is the chief obstacle; it not acting like a freely pliant curtain, and going back over the posterior nares as it should.

If these cases could be operated upon in infancy, before the child had learned to talk, there would be more hope for the final acquisition of fair speech.

A remarkable case bearing upon this point, was that of a a young lady, who previous to the operation, tolerated the greatest freedom in the manipulation of the parts. The operation was a fine success. The parts became unusually pliable. The patient was a young lady of intelligence and ambition, exceedingly anxious to cultivate the voice, as she had a talent for music, and had already attained to a degree of proficiency, in voice and pronunciation, rarely met with in this class of patients.

The friends of the patient think that there has been improvement in the voice from the operation, but our observation of the case inclines us to think that there has been little or none.

Now, with a case so apparently favorable as was this, resulting in no improvement, the previous experience with this class of difficulties is borne out.

The patient has been advised not to attempt the operation for cleft palate, but to have the hare lip operated upon.

The patient was now anæsthetized, and an incision was made which commenced a few lines above the retreating angle of the slit, and terminated by paring the edge of the lip on the left of the slit. A second incision was then made, commencing at the first one, near its origin, and terminating by cutting a flap in the lip on the right side of the slit.

After hemorrhage had ceased, the parts were nicely approximated, the flap in the right side of lip brought down, and used as a border for the lip, and the whole secured by several large and small waxed, silken sutures.

This restored symmetry to the lips, which, when the patient has grown a good moustache, will give a normal appearance to this part of his features.

Adhesive straps were applied to the wound, passing back as far as the ears, on either side, and holding in their grasp the folds of the cheeks, which are pressed towards the median line, thus removing extension from the wound.

The stitches will be removed in five or six days, the parts in the meantime to be kept as quiet as possible.

---

### *Typical Case of Cystic Degeneration of Inferior Maxilla.*

Samuel M., English, aged 43 years, farmer. Fourteen years ago, the patient who is a well-nourished, robust man, received a blow on the side of the jaw, from a horses' head, after which a small "gum boil," as he expressed it, commenced growing. It is painless.

*On examination*, find a globular, elastic tumor projecting upon the cheek and into the mouth, which is connected with the lower jaw. The neighboring glands are not affected.

An exploring needle has been used before bringing the patient to the clinic and, "cystic disease of the jaw" diagnosed. Whether it originated in a tooth cyst, or at the bottom of the alveolar process, cannot be said. It is proposed to remove the walls of the cyst proper, and all degenerated tissue connected with it, leaving the base of the jaw intact, thus avoiding a more radical operation and preserving the integrity of the jaw.

The patient being anæsthetized, the teeth involved in the diseased portion were extracted, and the knife and bone-gnawing forceps used, to remove all the degenerated mass. The base of the jaw was left intact, and its firm outline could be felt by the fingers, after the removal of the diseased structure.

The cyst proved to be binocular and communicating, having a ridge of bone between the two portions.

Boracic acid dressings were ordered.

